# Contrast-enhanced CT for attenuation correction in ultra-high sensitivity long-axial field-of-view lymphoma PET: amplified quantification problems?

**DOI:** 10.1186/s40658-026-00866-4

**Published:** 2026-04-13

**Authors:** Jan-Luca Penner, August Blomgren, Alexander Weissensee, Michelle Amon, Shayan Acikgöz, Clemens Mingels, Justin Ferdinandus, Foroud Aghapour Zangeneh, Urban Novak, Thomas Pabst, Axel Rominger, Kuangyu Shi, Hasan Sari, Robert Seifert

**Affiliations:** 1 Department of Nuclear Medicine, University Hospital Bern, Inselspital, University of Bern, Rosenbühlgasse 25/27, CH-3010 Bern, Switzerland; 2 Department I of Internal Medicine, Center for Integrated Oncology Aachen Bonn Cologne Duesseldorf, University of Cologne, Medical Faculty and University Hospital Cologne, Cologne, Germany; 3 Department of Medical Oncology, Inselspital, University Hospital Bern, University of Bern, Bern, Switzerland; 4 Siemens Healthineers International AG, Zurich, Switzerland

**Keywords:** Lymphoma, PET, PET/CT, FDG-PET/CT, FDG, Deauville, LAFOV, LAFOV-PET, LAFOV-PET/CT, Attenuation correction

## Abstract

**Purpose:**

It is unclear whether the use of contrast enhanced (CE) CT for attenuation correction (AC) of [^18^F]FDG PET leads to higher quantification inaccuracies when used in high-sensitivity LAFOV systems. This project aimed to assess the clinical feasibility of CE-CT for AC in LAFOV-PET for lymphoma patients.

**Methods:**

Lymphoma patients who underwent LAFOV-[^18^F]FDG-PET for restaging with low dose AC-CT and diagnostic CE-CT were included in this retrospective analysis. PET images were reconstructed using ultra-high sensitivity (UHS) mode with CE-CT and AC-CT. Lesions and reference regions (liver and mediastinal blood pool (BP)) were evaluated.

**Results:**

SUV_max_ of BP and the liver increased when CE-CT was used instead of AC-CT for AC (BP: median 2.41vs.2.21, *p* < 0.01, 8.78% intra-patient increase; liver: 3.07vs.2.87, *p* < 0.01, 7.86% medina intra-patient increase). Similarly, SUV_mean_ was higher in CE-CT reconstructions (BP: 1.96vs.1.77, *p* < 0.01, 10.14% median intra-patient increase; liver: 2.50vs.2.32, *p* < 0.01, 7.54% median intra-patient increase). SUV_max_ of lesions showed a similar magnitude of increase (5.97vs.5.68, *p* < 0.01, 5.80% median intra-lesion increase). The SUV-ratio of the lesions to reference organs decreased when CE-CT instead of AC-CT was used for AC (BP: 2.79vs.2.99, *p* < 0.01, median per-lesion decrease − 4.90%; liver: 1.85vs.1.97, *p* < 0.01, median per-lesion decrease − 2.64%).

**Conclusion:**

The use of CE-CT for AC of LAFOV-PET in UHS mode leads to higher SUV measurements. Also, lymphoma lesions show a consistent increase in uptake. The errors in the lesions seem higher than in conventional standard axial field-of-view PETs that use CE-CT for AC. Therefore, interpretation of borderline cases warrants attention to potential errors.

**Supplementary Information:**

The online version contains supplementary material available at 10.1186/s40658-026-00866-4.

## Introduction

2-[^18^F]fluoro-2-deoxy-D-glucose positron emission tomography ([^18^F]FDG-PET) is routinely used for initial staging as well as treatment response evaluation in lymphoma patients. Several prospective trials, such as HD18 and HD21, used [^18^F]FDG-PET to guide therapy de-escalation for advanced Hodgkin lymphoma stages [1, 2]. For diffuse large B-cell lymphoma (DLBCL), prospective trials like PETAL also included [^18^F]FDG-PET to detect treatment failure [3]. On this basis, current clinical guidelines like ESMO recommend [^18^F]FDG-PET staging for diffuse large B-cell lymphoma (DLBCL) and Hodgkin lymphoma [4, 5].

Long axial field-of-view (LAFOV) positron emission tomography (PET) is a recent advancement in PET scanner technology that enables highly sensitive detection of tracer accumulations [6, 7]. With greatly improved sensitivity, they enable a reduction of administered tracer activity, scan time and increased image quality [8, 9]. LAFOV-PET evaluation or preparatory simulations have been done for several PET vendors because of those advantages [10–12]. However, LAFOV-PET also poses challenges for image reconstruction, like more oblique angles for coincidence lines of response (LOR). Along a LOR in a PET scan, the attenuation factor (AF), is determined by an attenuation correction computed tomography (AC-CT), is used to estimate the fraction of photons expected to reach the PET-detectors [13, 14]. Longer LORs are subject to more attenuation due to more tissue that photons must pass through. This may make LAFOV-PET scans especially susceptible to inaccurate AF. This is especially the case for the ultra-high sensitivity mode (UHS), which is used clinically and uses the oblique LOR with an acceptance angle of 52° in the PET reconstruction [11, 15].

For the staging of lymphoma patients in our clinic, contrast-enhanced CT in the portal venous phase (CE-CT) and PET are often acquired subsequently in one examination. This allows for a combined morphologic and metabolic assessment of stage and response. However, if the CE-CT is used for AC of the PET image, the AFs might be overestimated, leading to an artificially increased uptake in the PET images [16–22]. A previous study on Hodgkin patients investigated the attenuation correction of conventional PET scans and found that the standardized uptake value (SUV) in tumors and lymphoma lesions increased when AC was done with CE-CT (e.g. SUV_max_ in liver, blood pool and lymphoma tissue increased by 5–13% on average in UHD reconstructed images); the authors concluded that the change was not clinically relevant [21]. Still, the same study showed slightly larger influences on liver and blood pool compared to lymphoma lesions on [^18^F]FDG-PET [21]. However, to date, the impact of using CE-CT for AC for LAFOV-PET remains unclear. This is especially relevant for lymphoma patients where the reference organs, such as liver and blood pool, show high contrast agent accumulation and can be more susceptible to errors in AFs when used for AC-CT.

Therefore, this project aimed to investigate the clinical feasibility of CE-CT for attenuation correction in LAFOV-PET of lymphoma patients. Changes in uptake values in both lymphoma lesions and reference organs were assessed in patients referred for restaging PET to evaluate treatment response.

## Methods

### Image acquisition

A Biograph Vision Quadra LAFOV-PET/CT Scanner (Siemens Healthineers, Erlangen, Germany) at the Department of Nuclear Medicine at the University Hospital of Bern was used for image acquisition. The study included all patients who underwent [^18^F]FDG-PET between January and March 2022, had an AC-CT and a CE‑CT acquired during the same examination, and provided general research consent (*n* = 30). Of these, reconstruction using AC-CT and CE-CT was technically feasible in a subset (*n* = 21), which were included in the analysis. Ethical approval of the local ethics committee for the retrospective analysis was obtained (KEK 2022 − 00486). Patients were injected with [^18^F]FDG (3 MBq per kg bodyweight, Median injected activity 210 MBq, Q1-Q3 192–259 MBq) and scanned with arms up, in a head-first supine position. A topogram was acquired from skull to mid-thigh. Two CT scans were performed: a low-dose AC-CT that covered the same range as the PET scan and a CE-CT in which the neurocranium was partially spared in patients with no known or suspected involvement of the central nervous system to reduce the absorbed dose of the eye lenses. The AC-CT was done before PET acquisition, whereas the CE-CT was performed afterwards. The acquisition and reconstruction parameters for the CT images can be found in Table 1. For the CE-CT, a standard amount of injected contrast medium was used (Iomeron 400 mg/ml or Ultravist 370 mg/ml was diluted to a concentration of 80% with saline solution (sodium chloride 0.9%), 120 ml was given to each patient). Injection speed varied, e.g. with respect to the caliber of the venous catheter in use. The CE-CT acquisition was done 70 s after injection of the contrast medium. In the patients with missing coverage of the skull in the CE-CT, the missing section was replaced with the corresponding part of the AC-CT images. This was achieved by co-registering the AC-CT and CE-CT scans using a combination of rigid and non-rigid registrations, and by filling the missing CE-CT slices with the corresponding slices from the AC-CT scans. PET images were reconstructed separately using AC-CT and CE-CT for attenuation correction.

**Table 1:** – Parameters used for the acquisitions and reconstructions of the CT images

Parameter	AC-CT	CECT
Range Care kV ref. kV (Mean)	100–120 (115)	80–120 (101)
Range CareDose ref. mAs (Mean)	35–60 (41)	100–307 (146)
Pitch	1.5	1
Rotation time (s)	0.33	0.5
Matrix size	512 × 512	512 × 512
Pixel size (mm)	1.52 × 1.52	0.98 × 0.98
Slice thickness	3	2
Slice increment	1.65	2
Kernel	Br38	Br32

### Image analysis

Segmentation of the lymphoma lesions and reference regions (liver, blood pool) was done using PMOD (Version 4.2, Bruker Corporation). Measurements of SUV_max_ were done by manually defined spheroid volumes-of-interest (VOIs) in the reference regions, liver and lumen of the mediastinal aorta (representing the blood pool). Mean VOI volumes were 60.2 ccm for liver (Q1-Q3 33.5–113.0), and 3.1 ccm for aorta (Q1-Q3 1.4–4.2). For lesion segmentation, isocontour VOIs with a 41% SUV_max_ threshold were defined in the PET image reconstructed with the AC-CT. Alternatively, e.g., in case of low uptake compared to surrounding tissue, smaller VOIs were placed in sites of residual lymphoma tissue. In case of extensive disease, a subset of lesions was measured to obtain a representative sample. When former lymphoma lesions could not be visually delineated from the surrounding tissue on the interim PET or the corresponding CT, no VOI was placed. All VOIs were copied from the AC-CT reconstructed PET onto the CE-CT reconstructed PET instead of defining separate VOIs for each reconstruction, as SUV_max_ is less sensitive to the exact VOI delineation.

TotalSegmentator was used to segment organs on both CT images [23]. The organ masks were used to extract the mean linear attenuation coefficient of each organ of interest from the attenuation maps corresponding to the different CT images. The aorta and liver were chosen as they are reference organs for the Deauville score evaluation. The L5 vertebra and right gluteal muscle was chosen to estimate the attenuation changes in bone and muscle tissue, where lower contrast medium accumulation was expected.

### Statistical analysis

Statistical analysis was performed using R statistical analysis software (version 4.4.2) [24]. Histograms of SUV_max_ distributions were created to compare results from the CE-CT and AC-CT-based PET reconstructions. Probability density functions for the distributions were calculated to complement the histograms. The intra-patient and intra-tumor difference in SUV was calculated for reference tissues and lesions, respectively. SUV ratios (SUVR) were calculated for all tumors and reference tissues for both PET reconstructions. Histograms with complementary PDFs were generated, similar to for the SUV_max_ data, and intra-lesion changes in SUVR were calculated. Differences in the SUVR distributions between the image reconstructions were tested for significance using the Wilcoxon signed ranked test, using *p* < 0.05 as threshold for statistical significance. Each lesion was assigned a Deauville score (Deaville 2, if SUVR^aorta^ ≤ 1; Deauville 3, if SUVR^aorta^ > 1 and SUVR^liver^ ≤ 1, or Deauville 4, if SUVR^liver^ > 1). Deauville score 1 and 5 were left out of the analysis since they at least partly require comparison to a pervious examination, whereas this study only considers changes of SUVR for a given examination. The score was assigned only using the measured values in the VOI, not accounting for reading physicians’ assessment. The SUVR from the AC-CT and CE-CT reconstructed PET images were correlated by linear fitting. The uncertainty in the linear fit was used to determine the uncertainty in the Deauville score, which was used to calculate the probability of a Deauville misclassification at each SUVR when the CE-CT is employed for attenuation correction. See the supplemental text and supplemental Figs. 1 and 2 for further details on the lesion-wise Deauville error estimation.

## Results

### Patient characteristics

Of the 21 restaging [^18^F]FDG-PET examinations, 18 patients presented measurable lymphoma lesions. In the other three patients, only the reference organs were analyzed because no clearly delineable lymphoma manifestation was left at restaging. Of the patients with measurable lesions, eight had at least one lesion with a Deauville score of 4 or 5. Most patients were referred for restaging of DLBCL or Hodgkin lymphoma (see supplement Table 1 for details).

### Changes in the attenuation maps

**Fig. 1:**
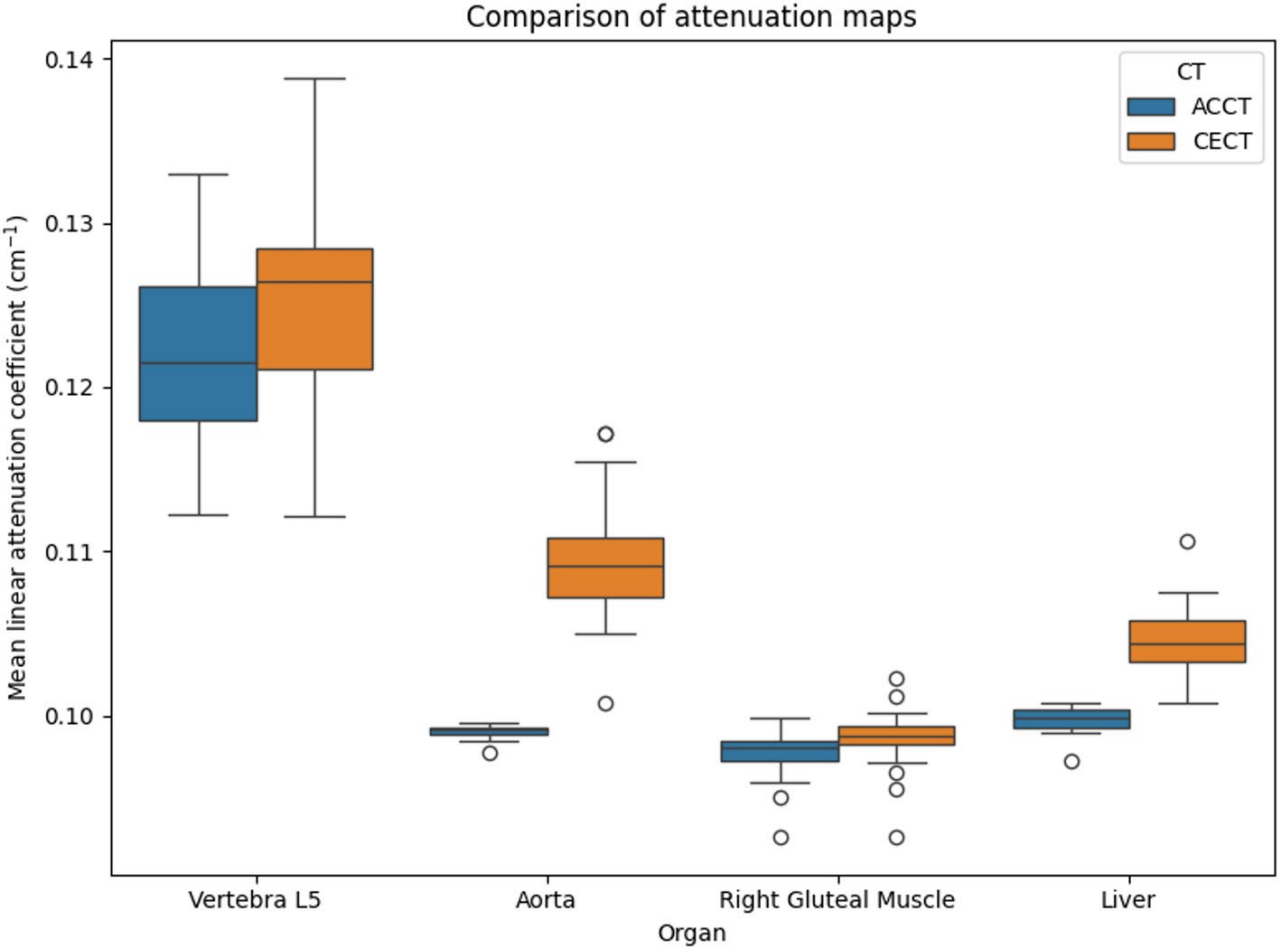
Box plots of the mean linear attenuation coefficients in different tissues for both attenuation maps. The largest increase is found in the aorta, followed by the liver, L5 vertebra and gluteal muscle. The whiskers end at Q1-1.5 IQR and Q3 + 1.5 IQR, respectively

The linear attenuation coefficient increased in every organ and patient measured, except for one case where the attenuation in the L5 vertebra decreased by less than 0.15%. The largest increase was found in the aorta, where the median increased by 10.4% when the CECT was used to generate the attenuation maps. The increase in the median in the liver was 4.9%. The results can be found as box plots in Fig. 1 and tabulated in Table 2.

**Table 2:** Values of the linear attenuation coefficient in different tissues accompanying the box plots in Fig. 1

Organ	CT	Median	Q1	Q3	Whisker low	Whisker high
Aorta	AC-CT	0.0992	0.0989	0.0993	0.0985	0.0996
	CE-CT	0.1091	0.1072	0.1109	0.1050	0.1154
Right gluteal muscle	AC-CT	0.0981	0.0973	0.0984	0.0959	0.0999
	CE-CT	0.0988	0.0983	0.0993	0.0971	0.1002
Liver	AC-CT	0.0998	0.0993	0.1004	0.0989	0.1008
	CE-CT	0.1044	0.1033	0.1058	0.1008	0.1076
Vertebra L5	AC-CT	0.1215	0.1180	0.1262	0.1123	0.1330
	CE-CT	0.1264	0.1211	0.1285	0.1121	0.1388

The whiskers end at Q1-1.5 IQR and Q3 + 1.5 IQR, respectively

### Effect on the physiological uptake of [^18^F]FDG in reference regions

**Fig. 2:**
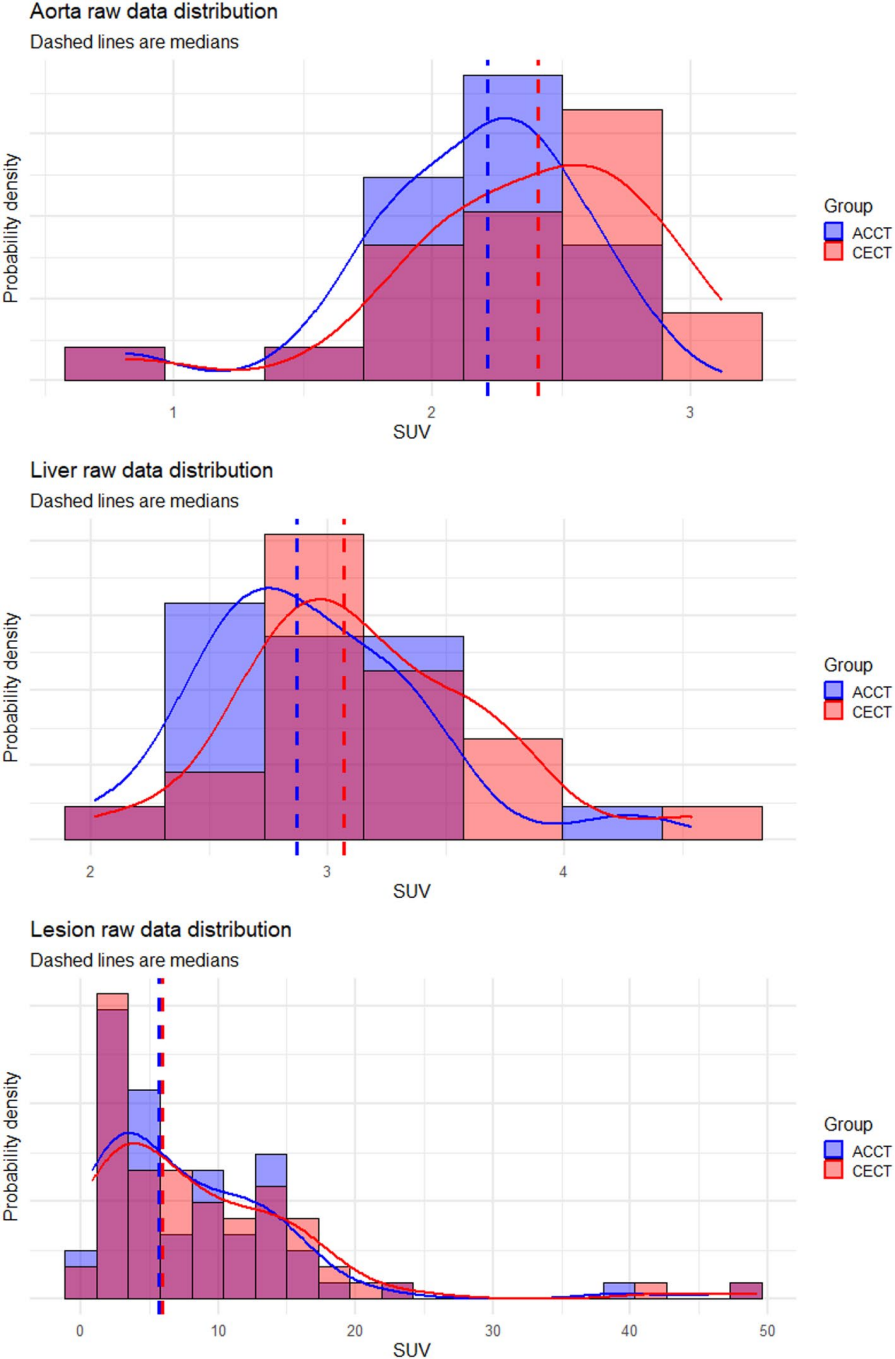
The distribution of SUVmax values in the reference tissues and lesions. Histograms are shown in blue for values obtained when the PET was reconstructed with AC-CT and in red for the CE-CT reconstruction, and purple where there is overlap. Solid lines on the histograms show the calculated probability-density functions for both reconstructions. Dashed lines show medians for each distribution

In the aorta, the measured SUV_max_ values were significantly higher when the CE-CT was used for PET reconstruction (median 2.41 vs. 2.21, *p* < 0.01; median intra-patient increase 8.78%). The same was true for SUV_mean_ (median 1.96 vs. 1.77, *p* < 0.01; median intra-patient increase 10.14%). Similarly, the SUV_max_ values in the liver were significantly higher in the PET images with CE-CT-based attenuation maps (median 3.07 vs. 2.87, *p* < 0.01; median intra-patient increase 7.86%), which was similar to the effect on SUV_mean_ (median 2.50 vs. 2.32, *p* < 0.01; median intra-patient increase 7.54%). For both reference tissues, SUV_max_ and SUV_mean_ increased in all patients when CE-CT was used for attenuation correction. The distributions displayed as histograms of measured SUV_max_ values for reference regions can be found in Fig. 2, with further details available in Table 3. In the reference tissues, the whole SUV distribution shifts towards higher values.

**Table 3:** Comparison of the uptake in the reference organs and lesions

SUV_max_ results	Aorta		Liver		Lesions	
Statistic	AC-CT	CE-CT	AC-CT	CE-CT	AC-CT	CE-CT
Median	2.21	2.41	2.87	3.07	5.68	5.97
MAD	0.45	0.50	0.48	0.49	5.81	5.98
IQR	0.54	0.70	0.64	0.70	9.73	10.63
Q1	1.88	2.03	2.60	2.82	2.53	2.71
Q3	2.41	2.73	3.24	3.52	12.26	13.34
SUV increases in cases	21/21 (100%)		21/21 (100%)		64/66 (97%)	
Median intra-patient change	8.78%		7.86%		5.80%	
Wilcoxon test p-value	4.8e-07		4.8e-07		2.3e-12	

### Effect on the uptake of [^18^F]FDG in lymphoma lesions

In the measured lymphoma lesions, the measured SUV_max_ was significantly higher when the CE-CT was used for attenuation correction (median 5.97 vs. 5.68, *p* < 0.01; median intra-lesion increase 5.80%), which was also the case for SUV_mean_ (median 3.89 vs. 3.67, *p* < 0.01, median intra-lesion increase 5.87%). Specifically, 97% of lesions showed this increase (64 out of 66). In the subset of lesions with a per-lesion Deauville score of 3 or 4 (*n* = 53), the SUV_max_ measurements showed a similar pattern (median 9.07 vs. 8.80, *p* < 0.01; median intra-lesion increase 5.80%). See Table 3 for details. The histogram of the SUV_max_ values can be found in Fig. 2. Similar to the reference tissues, the distribution shifts towards higher values, but the shift is less pronounced.

### Effect on the ratios of lesion to reference region (SUVR) on [^18^F]FDG-PET

**Fig. 3:**
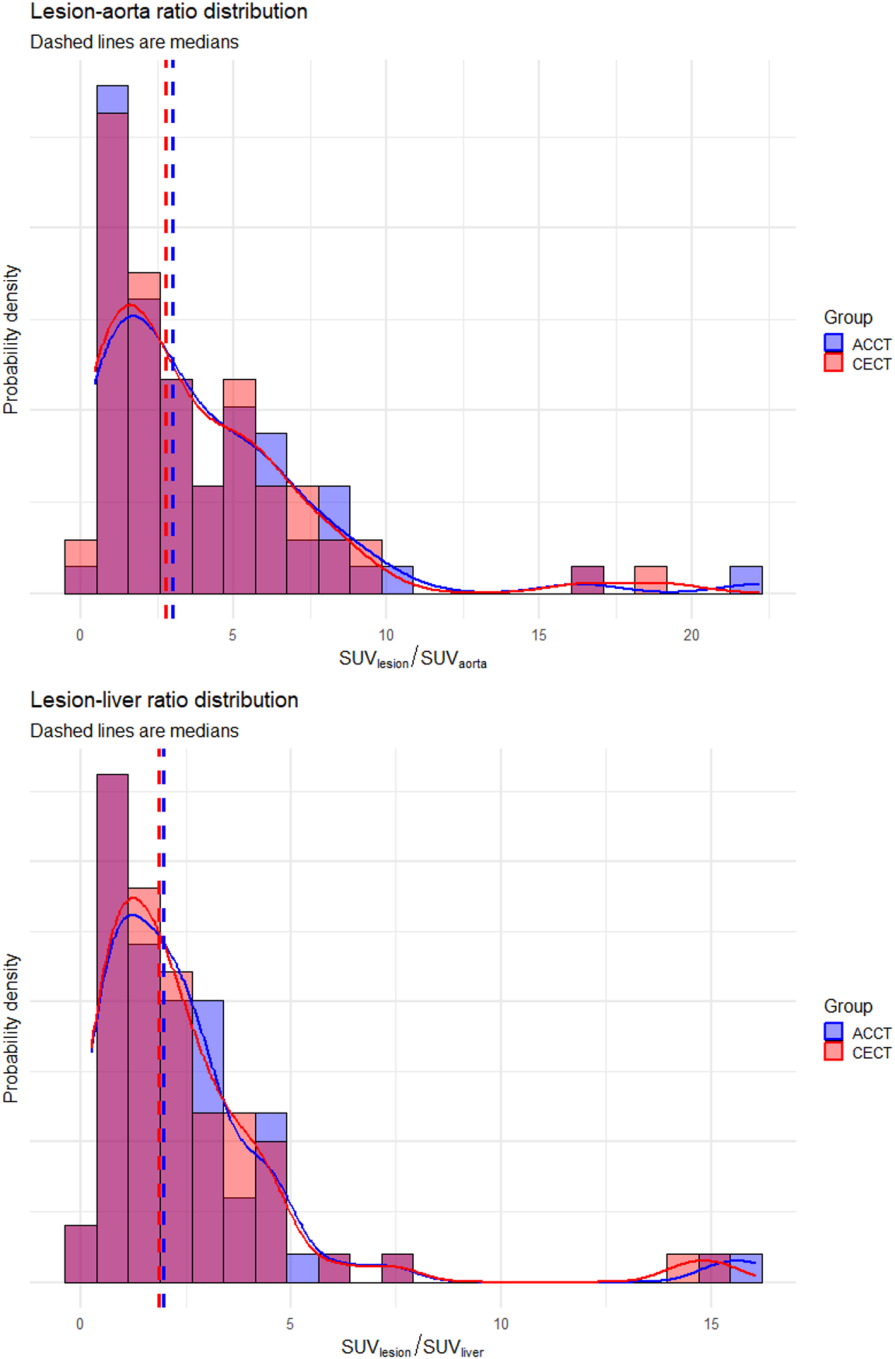
Distributions of SUV ratios of lesions to reference tissues. The distributions of the lesion to liver and lesion to aorta ratios are shown for the AC-CT and CE-CT reconstructions. There is a slight decrease in SUVR in most cases

**Fig. 4:**
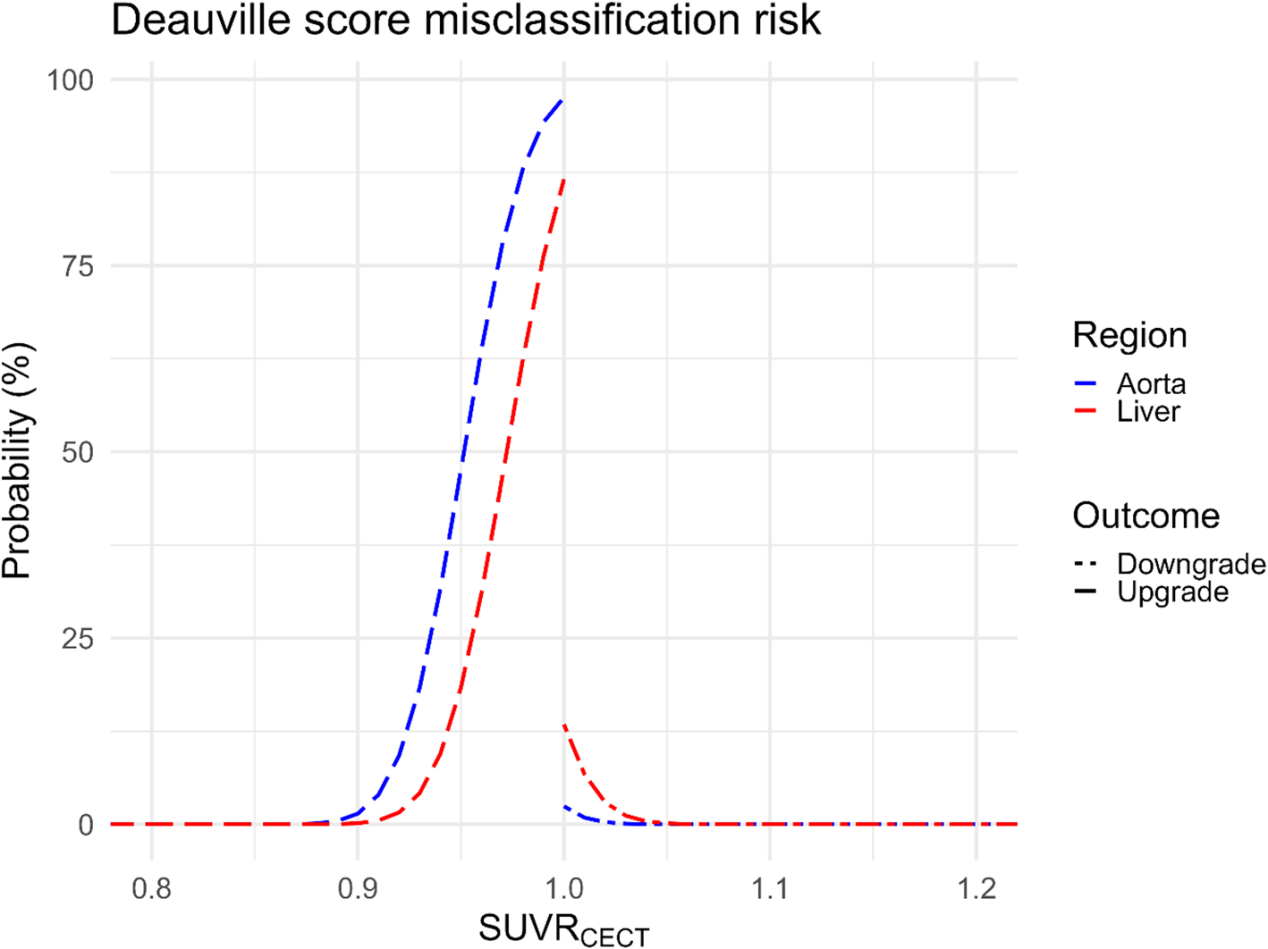
Calculated probabilities for misclassification of lesions relative to the reference tissue. Calculated probabilities for misclassification of lesions according to the Deauville score when the CE-CT is used for attenuation correction are shown. In the legend, the outcome “Downgrade” means that the observed SUVR_CE-CT_ is a false downgrade relative to the SUVR_AC-CT_. For a SUVR_CE-CT_ that approaches 1 from the upper limit, the probability of false upgrade is 2.5% in the aorta and 16% in the liver. Conversely, for a SUVR_CE-CT_ that approaches 1 from the lower limit, the probability of false downgrade is 97.5% in the aorta and 84% in the liver. The larges risk of misclassification occurs for SUVR_CE-CT_ values just below 1, as the risk of observing a false outcome is high. See supplement Fig. 2 for details

**Fig. 5:**
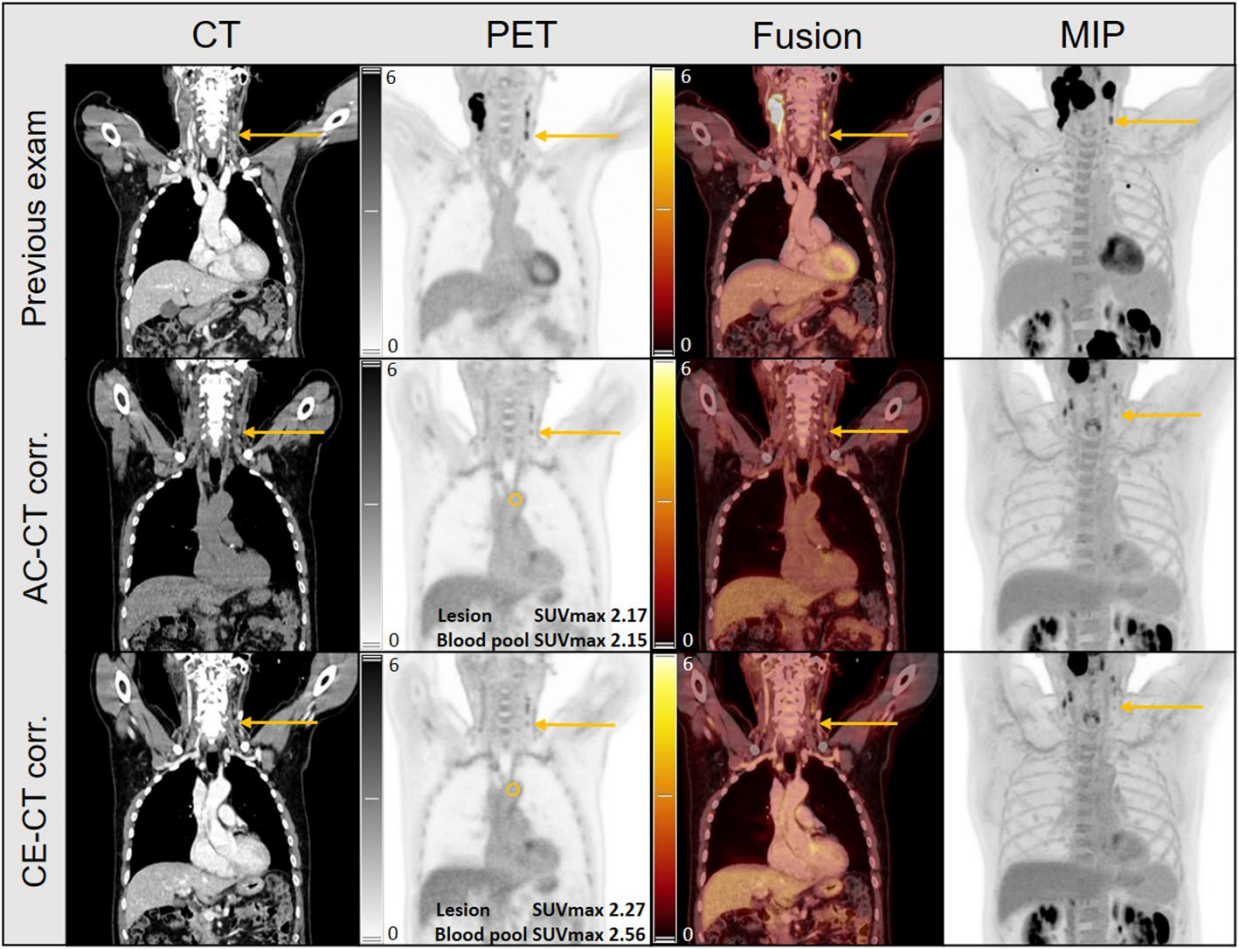
Higher mediastinal blood uptake with CE-CT PET reconstruction. Previous PET, restaging PET with AC-CT reconstruction and restaging PET with CE-CT reconstruction are shown. The marked lymphoma lesion (lymph node) showed an SUVmax higher than the blood pool when PET was reconstructed using CE-CT, whereas it was lower than the blood pool when reconstructed with AC-CT. An arrow indicates the lymph node, and the blood pool is outlined with a circle. PET window: SUV-bw 0–6; AC-CT window: center 40 HU, width 300 HU; CE-CT window (including CT of previous exam): center 45 HU, width 315 HU

The ratio of lesion SUV_max_ to liver (SUVR^liver^) was significantly lower when the CE-CT was used for attenuation correction (median 1.85 vs. 1.97, *p* < 0.01; median intra-lesion decrease − 2.64%) in 76% (50/66) of the lesions. Similarly, the ratio of lesion SUV_max_ to blood pool (SUVR^aorta^) was significantly lower when the CE-CT was used for attenuation correction (median 2.79 vs. 2.99, *p* < 0.01, median intra-lesion decrease − 4.90%) in 91% (60/66) of the lesions. The distribution of calculated SUVR for the lesions compared to different reference tissues using the two PET reconstructions can be found in Fig. 3; Table 4. The likelihood of assigning a lesion an incorrect Deauville grade, according to the linear model, is shown in Fig. 4. For an observed SUVR_CE-CT_ that approaches 1 from above, the probability of false upgrade is 2.5% in the aorta and 16% in the liver. Conversely, for a SUVR_CE-CT_ that approaches 1 from the below, the probability of false downgrade is 97.5% in the aorta and 84% in the liver. In an example case in Fig. 5, the lymphoma lesion’s SUV_max_ changed from 2.17 to 2.27, thereby falling above or below the blood pool, depending on the CT used for reconstruction.

**Table 4:** SUV ratios of lesions and reference tissues

SUVR results	Lesion/Aorta		Lesion/Liver	
Reconstruction CT	AC-CT	CE-CT	AC-CT	CE-CT
Median	2.99	2.79	1.97	1.85
MAD	3.05	2.85	1.61	1.60
IQR	4.26	4.25	2.10	2.11
Q1	1.23	1.17	0.97	0.96
Q3	5.50	5.41	3.07	3.07
SUVR decreases in cases:	60/66 (91%)		50/66 (76%)	
Median intra-lesion change	-4.90%		-2.64%	
Wilcoxon test p-value:	1.1e-08		2.5e-04	

*IQR* interquartile range, *MAD* median absolute deviation, *Q *Quartile

### Correlation between increased attenuation and SUV_max_

**Fig. 6:**
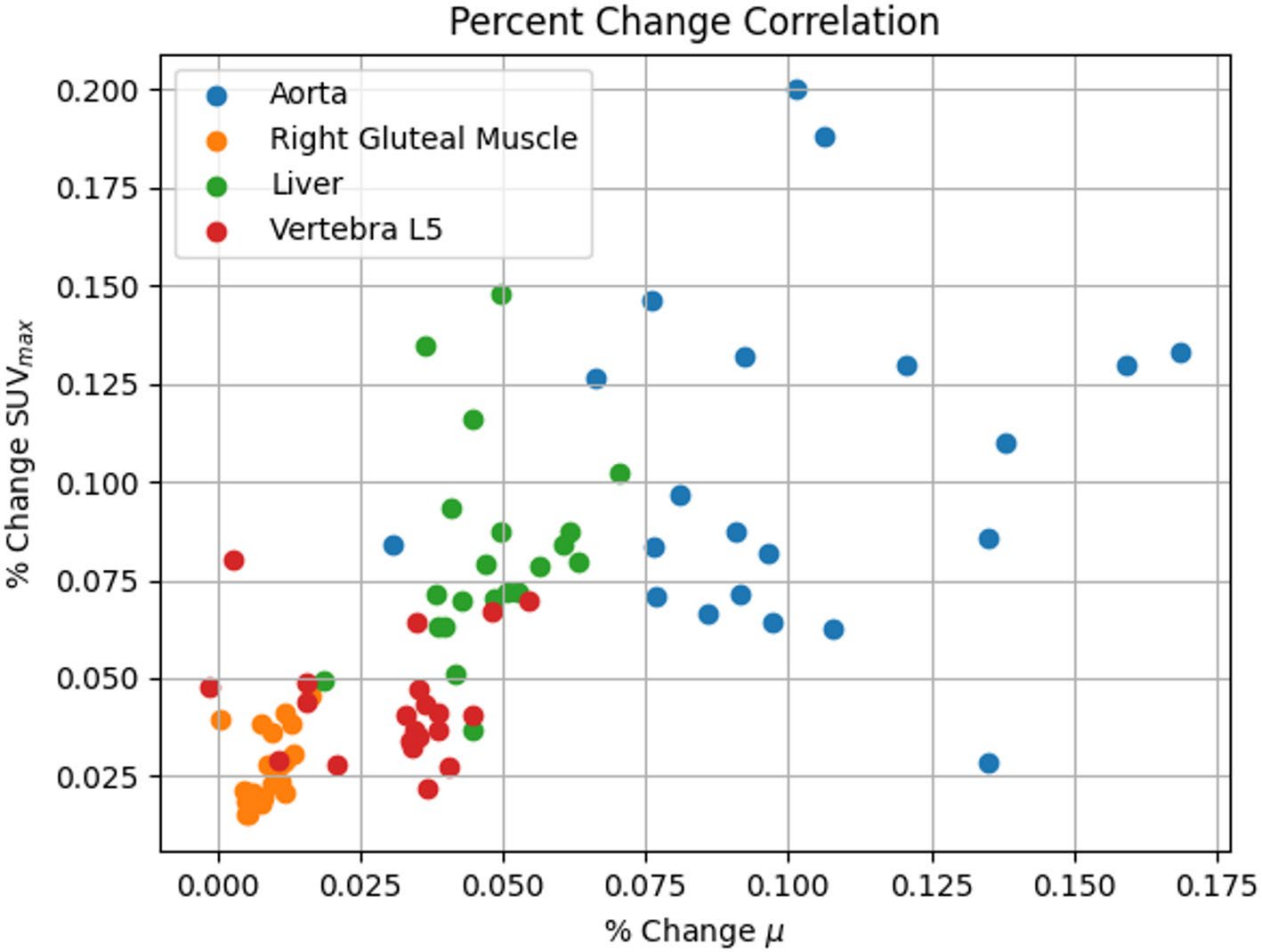
The correlation between the change of the linear attenuation coefficient and SUV_max_ when using the CE-CT for attenuation correction instead of the AC-CT. For each individual tissue, the correlation is not significant, but for the pooled data it is significant

The Pearson correlation and Spearman’s rank correlation tests were used to test if there was a significant correlation between the percentage change of the local linear attenuation coefficient and SUV_max_ in the aorta, liver, right gluteal muscle and the L5 vertebra. Tests were also performed on pooled data from all tissues. Figure 6 shows a scatter plot of the data used for the correlation testing, and the correlation results can be found in Table 5. For the correlations in individual organs, only Spearman’s test was statistically significant for the right gluteal muscle, although the correlation coefficient was only 0.535. When all data was pooled, both tests showed statistical significance, with Spearman having the highest correlation coefficient.

**Table 5:** Correlation of the change of linear attenuation coefficient and SUV_max_in different tissues

Tissue	Pearson		Spearman	
	*r*	*p*	*ρ*	*p*
Aorta	0.118	0.61	0.118	0.61
Liver	0.25	0.27	0.373	0.096
Right gluteal muscle	0.414	0.062	0.535	0.012
Vertebra L5	-0.045	0.84	-0.035	0.88
All	0.668	4*10^− 12^	0.744	5*10^− 16^

For the individual tissues, only the Spearman correlation test for the right gluteal muscle was significant, but the correlation was weak. When all data is pooled, both the Pearson and Spearman analysis found significant correlations

The data used for the correlations can be found in Fig. 5

## Discussion

In the present work, we analyzed the effect of using CE-CT vs. AC-CT for attenuation correction in PET image reconstruction on a LAFOV scanner, based on PET scans of lymphoma patients who were referred for restaging. When CE-CT scans were used for attenuation correction in LAFOV-PET, the uptake of both lesions and reference tissues showed elevated SUV values. However, as the median increase in SUV was larger in reference tissues than in the lesions, the ratio of lesion to reference tissue decreased. This indicates that the use of liver and blood pool reference organs on LAFOV-PET data could introduce systematic errors.

LAFOV-PET systems have higher sensitivity partly because of their increased axial field-of-view that allows the PET system to capture more data for reconstruction [6, 11]. The Quadra system (Siemens Healthineers, Erlangen, Germany) used in this study has an acceptance angle of 52°, compared to the e.g. 18° angle of comparable standard axial field-of-view (SAFOV) system (Biograph Vision 600 scanner, Siemens Healthineers, Erlangen, Germany) [11]. The greater acceptance angle enables a system sensitivity of 176 cps/kBq [11]. However, it can also introduce challenges in image reconstruction. First, the highest sensitivity is only achieved in the center of the scanner, from which annihilation photons could theoretically reach all detectors given the prespecified acceptance angle [11]. Secondly, the longer lines of coincidence lead to greater attenuation of annihilation photos, which is why accurate attenuation correction is especially relevant for them. The effect of contrast agent on attenuation maps could introduce larger errors compared to shorter lines of coincidence. Therefore, the use of CE-CT for attenuation correction could have larger errors in LAFOV PET than the already known deviations for conventional SAFOV PET.

Here, we could show that the median intra-lesion increase of SUV_max_ in lymphoma lesions was 5.8% when CE-CT was used for attenuation correction. This increase in line with the literature, with multiple studies reporting higher values when CE-CT was used for reconstruction [14, 15, 18]. Interestingly, the differences we observed in lymphoma lesions using the Quadra PET in the UHS mode are slightly higher than those described in the literature [17, 18, 21]. For example, a study using an analogue Biograph mCT Flow PET/CT scanner (Siemens Healthineers, Erlangen, Germany) found that applying CE-CT for AC resulted in only a 1.70% median intra-lesion increase in SUV_max_ in the hottest lesion of each patient [21]. The difference between these results and our observation can be partly explained by the lower in spatial resolution of the Biograph mCT compared to the Quadra, resulting in more partial-volume effects. Furthermore, the voxel size used in the study was not specified, but a larger voxel size would further add the partial volume effects, especially for small lesions. The spatial resolution of the scanner and the voxel size is not expected to change the results for the reference tissues to the same extent, because larger VOIs can be used. Consistent with this, our data for these regions appears to align with literature [21]. The higher median increase in uptake could also partly be attributed to the fact that LAFOV-PET operated in UHS mode has a higher acceptance angle and therefore AC artifacts could have a greater impact on quantification accuracy. Also, the difference could partly be explained by uncertainty due to the small patient cohorts that are used for their comparison with the analogue SAFOV system (*n* = 10) vs. the larger cohort of patients here (*n* = 18) for the LAFOV-PET [21].

In lymphoma PET imaging, the uptake of lymphoma manifestations is compared with reference uptake in the liver or blood pool to assess the vitality of residual disease [25]. A maximum Deauville score of 3 or lower (i.e. lesional uptake lower than liver) is regarded as sufficient response to treatment [26]. For example, in the treatment de-escalation of advanced Hodgkin disease, this assessment determines the need for additional therapy, which might cause long-term toxicity [27]. Given that the rate of Deauville positive findings is increasing over the study generations from HD18 to HD21, accurate knowledge of the limitations of the Deauville score in light of an ever-growing PET scanner sensitivity and resolution is of clinical relevance [1, 2]. Therefore, the assessment of CE-CT reconstruction on lesions and reference tissue is needed. Consistent with a previous report on CE-CT-based PET reconstruction in an analogue SAFOV system, which showed a 7.87% median SUV_max_ increase in the blood pool but only 1.7% in the hottest lesion of each patient (using UHD reconstruction), the present analysis found that quantification errors were more pronounced in reference tissues such as the liver and blood pool than in lesions [21]. This is expected, given the strong contrast agent accumulation in those reference tissues and thus the erroneously higher attenuation correction in those regions. Based on this, the ratio of lesion to background decreased when using CE-CT for attenuation correction, potentially impacting the Deauville rating in certain scenarios. Interestingly, since the quantification errors in the lesions increased more in our study than in literature, the increase was more similar to the reference tissues, resulting in smaller changes in SUVR. In our cohort, only two out of 66 lesions were downgraded by one Deauville score in a per-lesion-based analysis, as compared to two out of ten in another study [21]. The probability of the Deauville scores being artificially changed by the PET reconstruction with CE-CT was constrained to a span of SUVR that was smaller than the uncertainty in the underlying SUV values themselves [28]. Only the two lesions that were theoretically downgraded were in this span of SUVR_CE-CT_ values, where the risk of misclassification was above 5%.

In this work, SUV_max_ was used to assess potential reconstruction errors for several reasons. Firstly, it makes the results less sensitive to the delineation process, allowing us to delineate the lesions in one PET image and copy the VOIs to the other. Secondly, since small lesions suffer from low contrast recovery, SUV_max_ may be more representative of the true tracer concentration than SUV_mean_. However, for the reference tissues, where larger VOIs can be used, SUV_mean_ is expected to better represent the true tissue concentration, while SUV_max_ is expected to be biased to higher values. Furthermore, if the reference region is large and homogenous, SUV_mean_ will also be insensitive to the VOI delineation. To investigate the impact this may have, the analysis was performed again using SUV_max_ in lesions and SUV_mean_ in reference tissues. It was found that the median change of SUV in reference tissues was larger for SUV_mean_ than for SUV_max_, leading to a larger median change in the ratio of lesion to their reference tissues.

The correlation between the increase of attenuation and SUV_max_ was found to be weak. This was expected, as the AF for a given LOR is given by the line integral through the attenuation map along said LOR, meaning that the attenuation outside of the reference tissue also contributes to the AF. This means that not only does the increase of the attenuation coefficient in each voxel matter, but also the volume of the organs and their relative position to each other. This explains why the correlations are weak, even though both the local attenuation and the SUV increase in many cases.

One limitation of the study is the relatively small cohort of 18 patients with lesions (66 in total) and the small overall cohort of 21 patients with multiple different lymphoma entities. No clinically relevant changes of Deauville score were observed in this study, but a larger cohort is needed to assess the risk of clinically relevant changes. Another limitation is the mathematical approach of calculating the Deauville scores, which doesn’t account for the reading physician’s assessment of the patient. Moreover, low-dose-CT scans and CE-CT scans did not have an identical axial field of view. In the CE-CT, the cranium was not fully depicted (the scan regularly only included the base of the skull). The missing slices of the CE-CT had to be supplemented with low-dose CT slices. Also, patient motion between the acquisition of the CE-CT and AC-CT might result in bias due to different registration with the PET image. Future work may focus on the generation of virtual non-CE-CT for LAFOV-PET reconstruction to minimize the biases which have been described in this manuscript. Thereby, the acquisition of an additional low-dose AC-CT could be omitted to save time and radiation dose to the patient when CE-CT is acquired due to clinical indication. In addition, future work may expand to tracers other than [^18^F]FDG, as prostate-specific membrane antigen targeted PET also requires robust quantification when it is used in the context of radioligand therapy to quantify uptake, volume reduction, and to compare uptake with [^18^F]FDG in a dual tracer approach [29–31]. Since some protocols require certain SUV thresholds to establish an indication to treat patients, measurement deviation could be of relevance there as well [32].

## Conclusion

Attenuation correction with CE-CT compared to the standard low-dose AC-CT resulted in significantly higher SUV measurements in LAFOV [^18^F]FDG-PET of lymphoma patients. This was true for both lesions as well as reference tissues (liver and blood pool in the mediastinal aorta). The median SUV_max_ increase was larger in reference tissues compared to the lymphoma lesions. The errors in lymphoma lesions are higher than in conventional standard axial field-of-view PETs that use CE-CT for AC. Even though LAFOV-PET reconstruction with CE-CT is feasible, the potential quantification bias should be considered.

## Supplementary Information

Supplementary Material 1.

## Data Availability

The datasets generated during and/or analyzed during this study are not publicly available due to an identification risk.

## Declarations

### AI-assisted technologies and generative AI

Artificial intelligence (AI) tools were used to assist with translation, improving language, grammar, readability and literature search. The authors have reviewed and edited the content and take responsibility for it’s accuracy.
