# Supplementary material for: Contrast-enhanced CT for attenuation correction in ultra-high sensitivity long-axial field-of-view lymphoma PET: amplified quantification problems?
Source: EJNMMI Phys. 2026 Apr 13;13:50. doi: 10.1186/s40658-026-00866-4 (PMC13184045; doi:10.1186/s40658-026-00866-4)
Supplement: Supplementary file 1 — Supplementary Material 1. [file 40658_2026_866_MOESM1_ESM.docx]

# Supplement

## Details on the statistical Deauville error estimation

Since potential changes of the Deauville score (Supplement Fig. 1) were of interest when using the CE-CT for attenuation correction, special attention was paid to data close to the SUV ratio (SUVR) threshold of 1 in both reference tissues. Linear regression was performed with SUVR_ACCT_ as the independent variable and SUVR_CECT_ as the dependent variable. The model uncertainty was calculated by error propagation of the uncertainty in the model parameters:

Model: $y(x)=kx+m$,

model uncertainty: $u_{y}(x)=\sqrt{x^{2}u_{k}^{2}+u_{m}^{2}}$,

with $x={SUVR}_{ACCT}$ and $y$ being the predicted value of ${SUVR}_{CECT}$. Histograms of the residuals between the model and the data were created to check if the residuals were approximately Gaussian (Supplement Fig. 2). The model uncertainty was assumed to be Gaussian since the distribution of residuals was approximately Gaussian. This assumption allowed the use of a Gaussian cumulative density function (CDF) to calculate the total probability that a sample at any $x$ would result in $y\leq1$. That is, at every true SUVR_ACCT_, we can compute the probability of the SUVR_CECT_ being at most 1, which equivalent of calculating the probability that the Deauville score is different for the different reconstructions. For each $x$, the CDF was parametrized by $\mu=y\left( x \right)$ and ${\sigma=u}_{y}(x)$. For $x>1$, computing CDF($y=1$) is equivalent to calculating the probability that the Deauville score will be reduced if the CE-CT is used for attenuation correction. Conversely, for $x<1$, this is equivalent to calculating the probability that the Deauville score remains unchanged when the CE-CT is used for attenuation correction. Hence, the risk that the Deauville score is upgraded for a lesion in this region is 1 - CDF($y=1$).


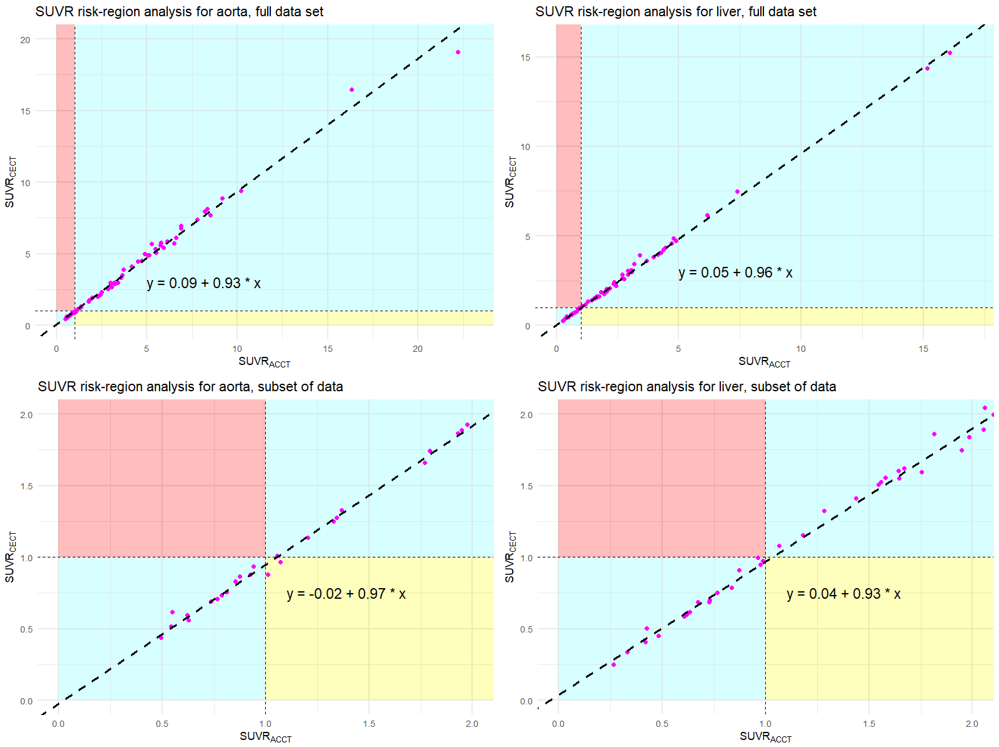


**Supplemental Fig. 1**

SUVR risk-region plots. Each panel has the SUVR_ACCT_ on the x-axis and SUVR_CECT_ on the y-axis. The blue regions show where the Deauville score is consistent between the two reconstructions. The yellow region shows where the Deauville score is falsely downgraded, and the red region shows where the Deauville score is falsely upgraded. The left column shows results for SUVR^aorta^, and the right column shows results for SUVR^liver^. The upper row shows the full data set while the lower panel is zoomed in on the region closest to the decision borders. Each panel has a linear fit to the visible data in the respective panel. Only in the bottom left panel, two lesions ending up in the downgrade region can be seen.

y)
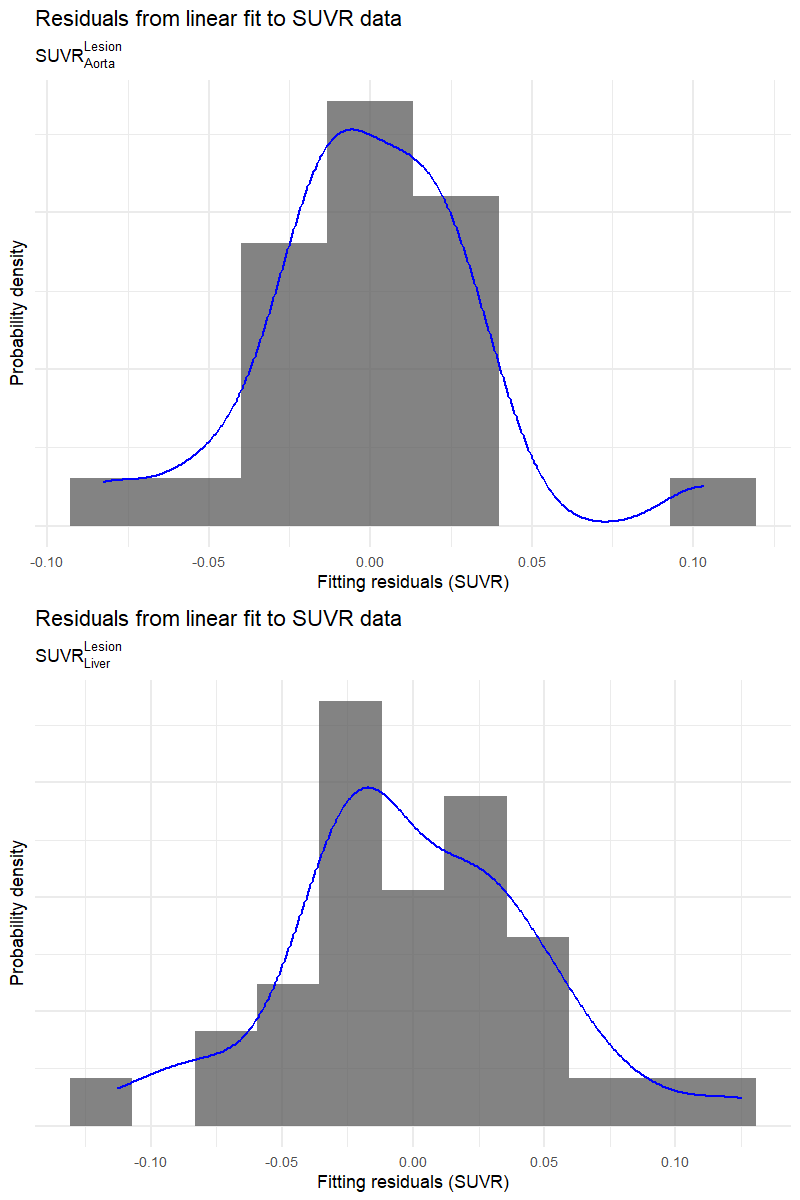


## Supplemental Fig. 2

Histograms of the residuals between the model fit and measured SUVR for both reference organs. The residuals of the lesion-aorta ratio are Gaussian shaped, whereas the residuals of the lesion-liver ratios are not as evidently Gaussian.

**Supplemental Table 1**

| ***DIAGNOSIS*** | ***PATIENTS (n)*** |
| --- | --- |
| ***DLBCL*** | *9* |
| ***Hodgkin’s lymphoma*** | *6* |
| ***Mantle cell lymphoma*** | *3* |
| ***Marginal zone lymphoma*** | *1* |
| ***Primary mediastinal BCL*** | *1* |
| ***Burkitt lymphoma*** | *1* |

Lymphoma subtypes in pat. population. DLBCL: Diffuse large B-cell lymphoma, BCL: B-cell lymphoma
